# PBK/TOPK inhibitor OTS964 resistance is mediated by ABCB1-dependent transport function in cancer: in vitro and in vivo study

**DOI:** 10.1186/s12943-022-01512-0

**Published:** 2022-02-08

**Authors:** Yuqi Yang, Qiu-Xu Teng, Zhuo-Xun Wu, Jing-Quan Wang, Zi-Ning Lei, Sabrina Lusvarghi, Suresh V. Ambudkar, Ning Ji, Zhe-Sheng Chen

**Affiliations:** 1 Department of Pharmaceutical Sciences, College of Pharmacy and Health Sciences, St. John’s University, Queens, New York City, NY 11439 USA; 2 Precision Medicine Center, The Seventh Affiliated Hospital, Sun Yat-Sen University, Shenzhen, 518107 Guangdong China; 3 Laboratory of Cell Biology, Center for Cancer Research, National Cancer Institute, NIH, Bethesda, 20892 USA; 4 Tianjin Medical University Cancer Institute and Hospital, National Clinical Research Center for Cancer, Tianjin’s Clinical Research Center for Cancer, Key Laboratory of Cancer Prevention and Therapy, Tianjin, 300060 China

**Keywords:** T-LAK cell-originated protein kinase (TOPK), PDZ-binding kinase (PBK), OTS964, ATP-binding cassette sub-family B member 1 (ABCB1), Multidrug resistance (MDR)

## Abstract

- ABCB1 overexpression significantly desensitized both drug-selected and gene-transfected cells, which overexpress ABCB1, to OTS964 and that this drug resistance can be antagonized by verapamil, a known ABCB1 inhibitor. Consistently, a similar trend was observed in tumor-bearing mice.
- OTS964 stimulated ATPase activity of ABCB1 and upregulated expression levels of ABCB1, resulting in induced resistance to other ABCB1 substrate-drugs, such as paclitaxel.
- OTS964 received a comparable affinity score and can dock into the substrate-binding site of human ABCB1 protein.

**Supplementary Information:**

The online version contains supplementary material available at 10.1186/s12943-022-01512-0.

## Aim

Accumulating reports have suggested that acquired drug resistance is linked to ATP-binding cassette sub-family B member 1 (ABCB1) overexpression. OTS964 is a potent inhibitor targeting to PDZ-binding kinase (PBK)/T-lymphokine-activated killer cell-originated protein kinase (TOPK). In present study, we aimed to explore the relationship between ABCB1 transporter and the regulation of OTS964 efficacy.

## Results interpretation and discussion

**Fig. 1:**
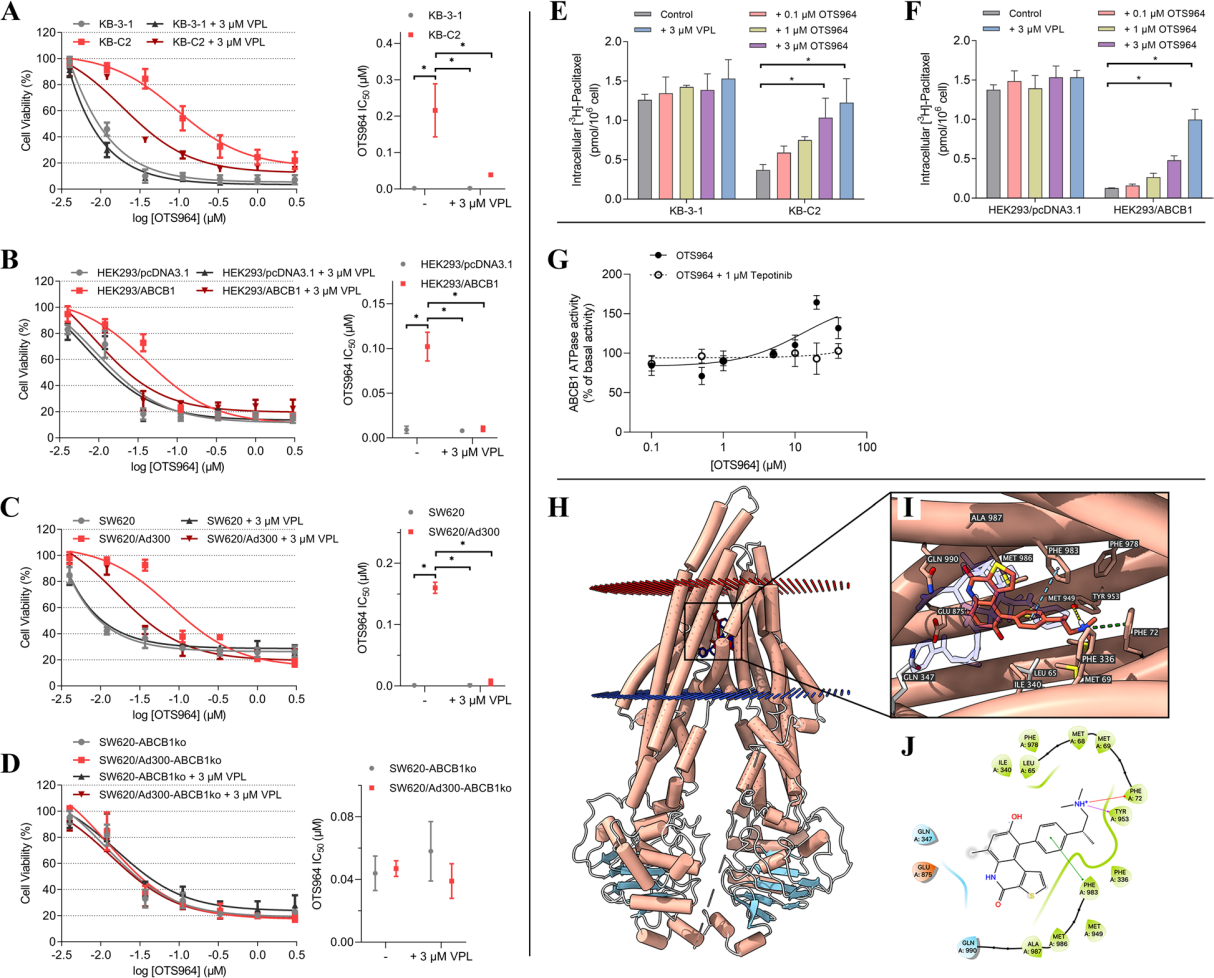
**A**-**D** Cytotoxic activity of OTS964 in drug-selected, gene-transfected, or gene-knockout cells and their respective parental cells. The concentration-response curves and IC_50_ values for OTS964 with or without a verified ABCB1 inhibitor in **A**) KB-C2 and KB-3-1, **B**) HEK293/ABCB1 and HEK293/pcDNA3.1, **C**) SW620/Ad300 and SW620, and **D**) SW620/Ad300-ABCB1ko and SW620-ABCB1ko cells. The GraphPad software [log (inhibitor) vs. response] was used to fit nonlinear regression and to calculate IC_50_ values. Each dot is expressed as mean ± SD from a representative of three independent experiments. *p < 0.05 versus the respective control group. **E**-**F** Effects of OTS964 on transport function mediated by ABCB1. The intracellular accumulation of [^3^H]-PTX in **E**) KB-C2 and KB-3-1 and **F**) HEK293/ABCB1 and HEK293/pcDNA3.1 cells after 2 h of pretreatment with vehicle, OTS964, or VPL. Data are expressed as mean ± SD from a representative of three independent experiments. *p < 0.05 versus the respective control group. **G** Effects of OTS964 on ATPase activity mediated by ABCB1. Effects of 0–40 μM OTS964 with or without 1 μM tepotinib on ATPase activity of ABCB1. Concentration of OTS964 was plotted against basal level (without OTS964) of ABCB1 ATPase activity. Data are expressed as mean ± SD from a representative of three independent experiments. **H**-**J** Highest-scoring docked pose of OTS964 within human ABCB1 at substrate-binding site. **H**) Overview of PTX and the best-scoring pose of OTS964 in the drug binding pocket of ABCB1 protein. PTX and OTS964 are displayed as colored sticks, blue: PTX; red: OTS964. **I**) Details of interactions between OTS964 and ABCB1 binding pocket. Predicted bonds are displayed as colored dash lines: hydrogen bond: yellow; *pi*-*pi* stacking: blue; cation-*pi* interaction: green. **J**) 2D OTS964-ABCB1 interaction. Important amino acids are displayed as colored bubbles (green: hydrophobic; blue: polar; red: positively charged). Predicted bonds are displayed as colored lines: green line: *pi*-*pi* stacking; purple line with arrow: hydrogen bond; red line: cation-*pi* interaction

By means of MTT-based cell viability assay, we examined the susceptibility of OTS964 to cells overexpressed ABCB1 and found that the effectiveness of OTS964 was restricted in both drug-selected and gene-transfected cells, which overexpress ABCB1, compared to those of the corresponding parental cells (Fig. 1A-C and Table S1). Interestingly, an ABCB1 inhibitor verapamil (VPL) can re-sensitize the acquired resistance to OTS964 and restore the efficacy of OTS964 to similar level as drug-sensitive cells do. Upon *ABCB1* gene knockout, the cell viability curves of drug-resistant cells were overlapping with those of the parental cells, and SW620/Ad300 cells became more sensitive to OTS964 after *ABCB1* knockout (Fig. 1C-D and Table S2). Meanwhile, doxorubicin (DOX), a verified ABCB1 substrate, was used as a reference to compare the degree of reduced efficacy caused by ABCB1-overexpressing (Fig. S1 and Table S1-S2). As we previously reported that OTS964 is a substrate-drug of ABCG2 [1], cells transfected with both transporters were used to confirm (Fig. S2). The cytotoxic activity of OTS964 was limited in B1/G2 cells relative to that in parental PEL cells, and that this effect can be partially re-sensitized by a known inhibitor of ABCB1 or ABCG2. Hence, we hypothesized that ABCB1 overexpression is possible to confer drug resistance to OTS964. Several mechanistic studies were subsequently performed to examine its possible mechanism.

To evaluate ABCB1-mediated transport function, a [^3^H]-PTX accumulation assay was conducted. As the cells were incubated with OTS964 for a short time, we supposed that OTS964 is unlikely to affect cell viability or other cellular functions, even though high concentrations were used. The results showed that, after co-treatment of PTX with high concentration (3 μM) of OTS964, an increased amount of PTX was detected in drug resistance cells but not in their corresponding parental cells (Fig. 1E and F). This effect may be the results of a high concentration of OTS964 competing with another substrate-drug PTX for transport function mediated by ABCB1, and thus leading to an enhanced intracellular accumulation of PTX. This assumption is further elucidated in our in silico molecular docking analysis.

As ABCB1 is an ATP-dependent transporter, stimulated ATP hydrolysis is generally coupled to substrate transport mediated by ABC transporters [2, 3]. To this end, an ATPase assay was performed to determine whether OTS964 can stimulate ABCB1 ATPase activity. We found that OTS964 concentration-dependently stimulates the vanadate-sensitive ABCB1 ATPase activity (Fig. 1G). The ATPase activity reached a peak of 164.3% of basal activity. Additionally, tepotinib, as an inhibitor of ABCB1 ATPase activity [4], can antagonize the stimulated ATPase activity and restore the ABCB1 ATPase activity to basal level. These results demonstrated that OTS964 may interact with the drug-binding domain of ABCB1 protein and behave as a substrate-drug of ABCB1 transporter.

The in silico molecular docking analysis is widely applied in the field of structural molecular biology as an efficient tool to predict ligand-protein interactions [5, 6]. In accordance with stimulatory results from ATPase assay, the docking simulation was conducted in the substrate-binding pocket (6QEX) of ABCB1. OTS964 received a docking score of − 7.2 kcal/mol. Moreover, our results showed that OTS964 is positioned in transmembrane region via hydrophobic interaction with amino acid residues and stabilized by *pi*-*pi* stacking interaction and cation-*pi* interaction formed with amino acid residues (Fig. 1H-J). To further validate the possibility that OTS964 may be a human ABCB1 substrate, we analyzed a verified ABCB1 substrate PTX under the same parameters (Fig. S3). Above results thereby demonstrated that OTS964 interacts with ligand-binding cavity of ABCB1 and behaves as a substrate for ABCB1 transporter.

**Fig. 2:**
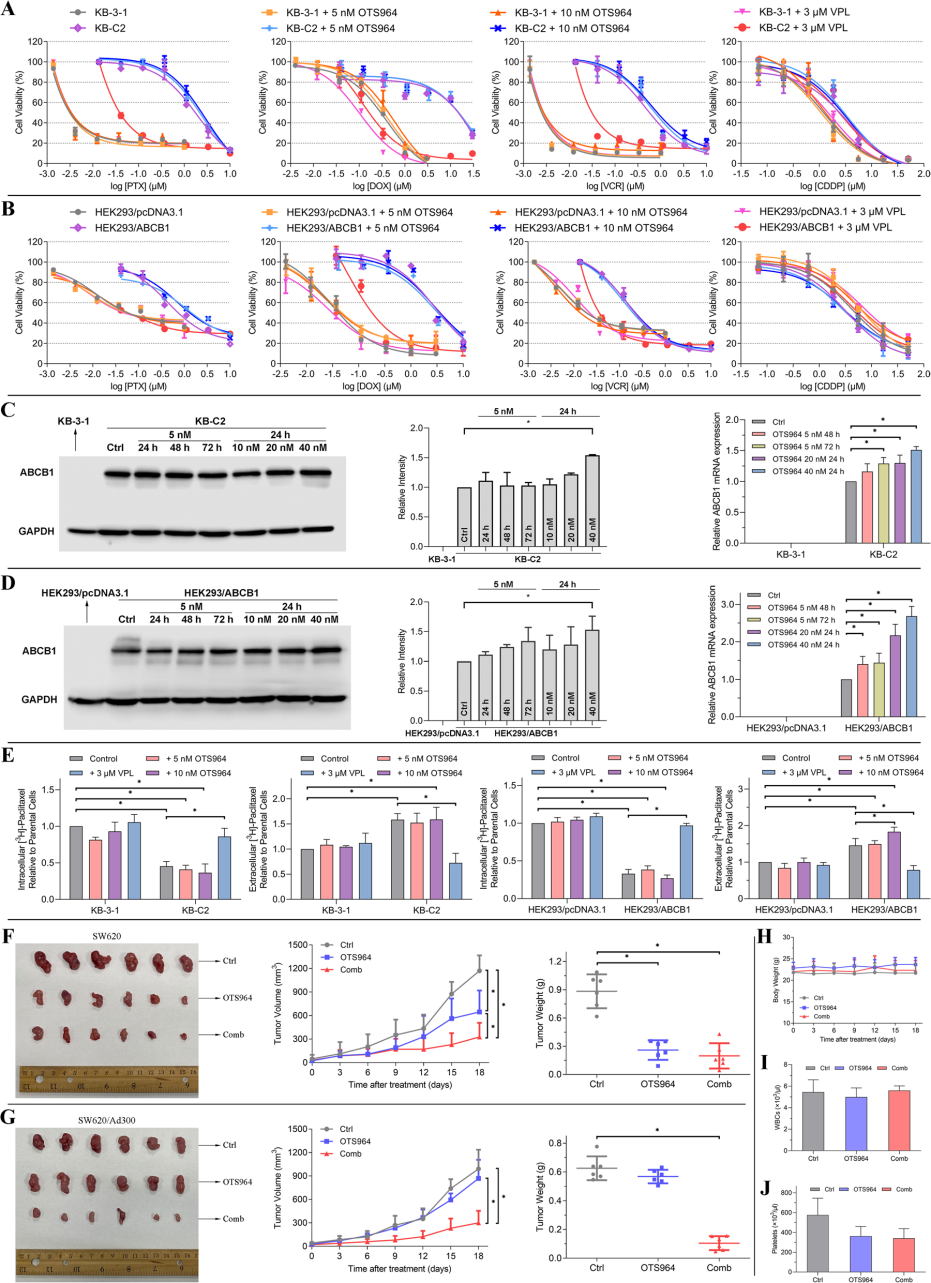
**A**-**B** Effects of OTS964 on the cytotoxicity of chemotherapeutic drugs in ABCB1-overexpressing cells. The effect of OTS964 on the cytotoxicity of PTX, DOX, VCR, and CDDP in **A**) KB-C2 and KB-3-1 cells and **B**) HEK293/ABCB1 and HEK293/pcDNA3.1 cells. The GraphPad software [log (inhibitor) vs. response] was used to fit nonlinear regression and to calculate IC_50_ values. In the concentration-response curve, each dot is expressed as mean ± SD from a representative of three independent experiments. **C**-**D** Effects of OTS964 on expression levels of ABCB1 protein and mRNA. The effect of OTS964 on ABCB1 protein and mRNA expression in **C**) KB-C2 and KB-3-1 cells and **D**) HEK293/ABCB1 and HEK293/pcDNA3.1 cells. In the Western blot analysis, the relative density of each protein band was analyzed by Fiji software, and ABCB1 protein expression levels were normalized to GAPDH before comparison. In the qRT-PCR analysis, data were calculated based on the comparative ΔΔC_T_ method and expressed as the relative fold changes. Data are expressed as mean ± SD from a representative of three independent experiments. *p < 0.05 versus the respective control group. **E** Effects of OTS964 on the intracellular and extracellular amount of [^3^H]-PTX in ABCB1-overexpressing cells after 72 h of treatment. The relative ratio of [^3^H]-PTX of KB-C2 and KB-3-1 cells, as well as HEK293/ABCB1 and HEK293/pcDNA3.1 cells in cell pellets or remaining medium after 72 h of treatment. The relative ratio was calculated as the treatment group divided by the parental control group. Data are expressed as mean ± SD from a representative of three independent experiments. *p < 0.05 versus the respective control group. **F**-**J** Antitumor activity of OTS964 in SW620 and SW620/Ad300 tumor xenograft models. **F**) From left to right: Images of excised SW620 tumor tissues from nude athymic mice at the end of treatment period. The changes of tumor volume in SW620 tumor xenograft model over time following the implantation. The weight of excised SW620 tumor tissues from nude athymic mice at the end of treatment period. **G**) From left to right: Images of excised SW620/Ad300 tumor tissues from nude athymic mice at the end of treatment period. The changes of tumor volume in SW620/Ad300 tumor xenograft model over time following the implantation. The weight of excised SW620/Ad300 tumor tissues from nude athymic mice at the end of treatment period. **H**) The changes of body weight during the treatment period. The **I**) WBC and **J**) platelet counts of nude athymic mice at the end of treatment period. Data are expressed as mean ± SD. *p < 0.05 versus the respective control group

Mechanistic studies have indicated that some chemosensitizers interact with the substrate-binding site of the transporter and compete with anticancer drugs for transportation, and thereby these modulators are themselves transported [7–9]. This enables a substrate-drug transported by ABCB1 can reposition as a competitive inhibitor to antagonize ABCB1-mediated drug resistance. To this end, a reversal study was conducted to evaluate whether OTS964 can be repurposed as a ABCB1 modulator. Low concentrations (5 and 10 nM), assuming non-toxic concentrations, of OTS964 were selected to rule out the possibility of additive toxicity. The results showed that OTS964 at low concentrations failed to restore drug sensitivity to ABCB1 substrate-drugs in drug-selected and gene-transfected ABCB1-overexpressing cells (Fig. 2A-B and Table S3). However, non-toxic OTS964 treatment promoted PTX resistance in resistant KB-C2 cells without affecting the PTX sensitivity in parental KB-3-1 cells. Consistently, a similar trend was observed in OTS964-treated ABCB1-transfected HEK293 cells. Moreover, it was found that the efficacy of non-substrate drug cisplatin (CDDP) was not significantly affected in cells treated with OTS964, suggesting that this effect may be specific to ABCB1. Of note, the exact nature of interaction between OTS964 and ABCB1 transporter, namely, whether OTS964 acts as a transported substrate or a competitive inhibitor, is dependent on the concentration used, the assay being used, and the varying activities of the transporters in each cell type. Therefore, our results do not warrant further testing of OTS964 as a chemosensitizer.

Since amplifying the ABCB1 drug efflux pump could be a possible mechanism for acquired drug resistance, a Western blot analysis was conducted to further evaluate the mechanism of action. Attempts to simulate the clinical settings and circumvent the off-target toxicity, we selected low concentration, assuming negligible cytotoxicity, for up to 72 h of exposure and high concentration, approached IC_50_ value, for 24 h of exposure. The results showed that, within 72 h, a low concentration (5 nM) of OTS964 did not significantly change the ABCB1 protein level in drug resistant cells overexpressed ABCB1 (Fig. 2C-D). After 40 nM of OTS964 incubated for 24 h, significantly stimulated ABCB1 protein expression was found in ABCB1-overexpressing cells. Given that ABCB1 protein upregulation could be in consequence of *ABCB1* gene amplification, we postulated that *ABCB1* mRNA level could be increased by OTS964. A qRT-PCR analysis was subsequently carried out. Remarkably upregulated *ABCB1* mRNA level was observed in resistant cells treated with OTS964 at low concentration (5 nM) with 72 h of incubation and at high concentrations (20 nM or 40 nM) with 24 h of incubation (Fig. 2C-D). Notably, there is a discrepancy between mRNA abundances and protein abundances especially for cells with 5 nM OTS964 treatment. Indeed, it has been shown that mRNA transcript abundances only partially, but not strongly, correlate with protein abundances, and the squared Pearson correlation coefficient is approximately 0.40 [10]. As above discrepant results are thought to be determined by cofactors, a detailed mechanism of this discrepancy and upregulation remains to be investigated further. Together, enhanced ABCB1 expression at protein and mRNA levels is an underlying reason for drug resistance induced by OTS964.

Combined the results from Western blot and qRT-PCR, we postulated that stimulated ABCB1 protein and mRNA allow a more functionable ABCB1 transporter to pump out its substrates, resulting in a decreased sensitivity to drugs transported by ABCB1. To further examine this assumption, a 72-h accumulation assay was conducted using the same concentrations as in reversal study, and the amounts of [^3^H]-PTX in both intracellular and extracellular space were quantified. Consistent with the cytotoxicity results, OTS964 at low concentration induced a reduction of PTX intracellular accumulation and an enhanced level of extracellular PTX in drug-resistant cells (Fig. 2E). Moreover, this effect was not observed in corresponding drug-sensitive cells. As ABCB1 protein expression is not significantly changed within 72 h exposure of OTS964 at low concentration, we postulated that OTS964 at negligible toxic concentrations may affect the transport function without altering the protein expression level. This hypothesis should be further verified. However, we noticed that, combined the results from 4-h accumulation assay and Western blot analysis, OTS964 at high concentration competitively inhibits PTX efflux leading to more PTX accumulating in drug-resistant cells, but paradoxically promotes ABCB1 expression at both transcriptional and translational levels. Therefore, we postulated that OTS964 at approached IC_50_ concentrations may have off-target activities in addition to its interaction with ABCB1 transporter, which needs to be collaborated further. It is also possible that OTS964 may induce a conformational change upon binding with ABCB1 protein, consequently preventing PTX binding and thus resulting in a competitive inhibitory of PTX efflux after short time exposure to OTS964. By contrast, after 72 h of OTS964 treatment, the stimulated ATPase activity, which would supply energy for efflux function, enables more PTX being pumped out from drug-resistant cells. This hypothesis remains to be further validated in the future.

Subsequently, to further translate our in vitro findings into in vivo evaluation, athymic nude mice were implanted with SW620 and its ABCB1-overexpressing SW620/Ad300 cells to establish tumor xenograft model. As a result, the tumor growth was significantly suppressed in OTS964-treated mice bearing SW620 tumors compared to the control group (Fig. 2F). By contrast, in SW620/Ad300 tumor-bearing mice, OTS964 alone treatment did not induce significant reduction in tumor size relative to those in the control group (Fig. 2G). These results suggested that the efficacy of OTS964 is restricted by ABCB1-mediated drug resistance. An immunohistochemistry analysis of ABCB1 from tumor xenograft model was also performed. Consistent with the results from Western blot, the expression level of ABCB1 protein was induced in OTS964-treated SW620/Ad300 tumor sections (data not shown). Interestingly, the combination of OTS964 and VPL significantly inhibited the tumor growth in SW620/Ad300 xenograft model compared to the control group, which, at least in part, verified that the limited efficacy of OTS964 is induced by ABCB1 overexpression (Fig. 2G). Given that toxicity is a major concern for any chemotherapeutic agent, the body weight of mice was used as an indicator of tolerability throughout the course of this study. Our results showed that no remarkable weight loss was caused by OTS964 either applied alone or used in combination with VPL (Fig. 2H). Also, the number of WBCs and platelets in all groups was within the normal range and without marked changes (Fig. 2I-J).

Collectively, this article presents in vitro and in vivo evidence that OTS964 is susceptible to ABCB1-mediated drug resistance and provides important indications for follow-up clinical use of OTS964.

## Supplementary Information

**Additional file 1.**

## Data Availability

The datasets used and/or analyzed during the current study are available from the corresponding author on reasonable request.

## Abbreviations

ABC transporter: ATP-binding cassette transporter
ABCB1: ATP-binding cassette sub-family B member 1
CDDP: Cisplatin
COL: Colchicine
DOX: Doxorubicin
MDR: Multidrug resistance
PBK: PDZ-binding kinase
PTX: Paclitaxel
qRT-PCR: Quantitative real-time PCR
TOPK: T-lymphokine-activated killer cell-originated protein kinase
VCR: Vincristine
VPL: Verapamil
WBCs: White blood cells
