# Supplementary material for: PBK/TOPK inhibitor OTS964 resistance is mediated by ABCB1-dependent transport function in cancer: in vitro and in vivo study
Source: Mol Cancer. 2022 Feb 8;21:40. doi: 10.1186/s12943-022-01512-0 (PMC8822834; doi:10.1186/s12943-022-01512-0)
Supplement: Supplementary file 1 — Additional file 1. [file 12943_2022_1512_MOESM1_ESM.docx]

**PBK/TOPK inhibitor OTS964 resistance is mediated by ABCB1-dependent transport function in cancer: *in vitro* and *in vivo* study**

Yuqi Yang^1^, Qiu-Xu Teng^1^, Zhuo-Xun Wu^1^, Jing-Quan Wang^1^, Zi-Ning Lei^1, 2^, Sabrina Lusvarghi^3^, Suresh V. Ambudkar^3^, Ning Ji^1, 4, *^, Zhe-Sheng Chen^1, *^

^1^ Department of Pharmaceutical Sciences, College of Pharmacy and Health Sciences, St. John's University, Queens, NY 11439, USA

^2^ Precision Medicine Center, The Seventh Affiliated Hospital, Sun Yat-Sen University, Shenzhen, Guangdong, 518107, China

^3^ Laboratory of Cell Biology, Center for Cancer Research, National Cancer Institute, NIH, Bethesda, 20892, USA

^4^ Tianjin Medical University Cancer Institute and Hospital, National Clinical Research Center for Cancer, Tianjin's Clinical Research Center for Cancer, Key Laboratory of Cancer Prevention and Therapy, Tianjin, 300060, China

^*^Corresponding author:

Zhe-Sheng Chen, Department of Pharmaceutical Sciences, College of Pharmacy and Health Sciences, St. John’s University, Queens, NY 11439, USA (Z.-S. Chen). E-mail: chenz@stjohns.edu.

Ning Ji, Tianjin Medical University Cancer Institute and Hospital, National Clinical Research Center for Cancer, Tianjin's Clinical Research Center for Cancer, Key Laboratory of Cancer Prevention and Therapy, Tianjin, 300060, China (N. Ji). E-mail: ningji@tmu.edu.cn.

**Contents for additional file**

[1. Background 3](#_Toc93769218)

[2. Material and methods 5](#_Toc93769219)

[2.1 Reagents, kits, apparatus, and software 5](#_Toc93769220)

[2.2 Cell lines and cell culture conditions 5](#_Toc93769221)

[2.3 Cell viability assay 6](#_Toc93769222)

[2.4 Accumulation assay 6](#_Toc93769223)

[2.5 ATPase assay 6](#_Toc93769224)

[2.6 Molecular docking analysis 7](#_Toc93769225)

[2.7 Western blot analysis 7](#_Toc93769226)

[2.8 Quantitative real-time PCR (qRT-PCR) 8](#_Toc93769227)

[2.9 Experimental animals and xenograft model 8](#_Toc93769228)

[2.10 Statistics 10](#_Toc93769229)

[3. Study design 10](#_Toc93769230)

[4. References for additional file 11](#_Toc93769231)

[Figure S1 14](#_Toc93769232)

[Figure S2 15](#_Toc93769233)

[Figure S3 16](#_Toc93769234)

[Table S1 17](#_Toc93769235)

[Table S2 17](#_Toc93769236)

[Table S3 18](#_Toc93769237)

[Table S4. 20](#_Toc93769238)

#

# 1. Background

Cancer is a major public health problem in many countries and has a trend to become the most serious life-threatening disease [1]. Many oncology drugs have been developed over the past decades to attack cancer cells in regulating cancer cell growth and survival. However, accumulating evidence has suggested that the clinical effect is limited and cannot last for a long time period due to the acquired resistance of tumor cells [2]. Overexpression of multidrug resistance (MDR)-associated ATP-binding cassette (ABC) transporters remain one of the major obstacles for cancer chemotherapy and induce acquired drug resistance [3]. Hitherto, 49 human ABC transporters (there appear to be 48 functional proteins) have been identified and can be further classified into 7 subfamilies of proteins, from ABCA to ABCG [4]. Among them, ATP-binding cassette sub-family B member 1 (ABCB1, P-glycoprotein/P-gp, multidrug resistance 1/MDR1), encoded by *ABCB1* gene maps on chromosomal locus 7p21, has a crucial role in protecting cells from endogenous and exogenous toxicants [5-7]. As a gatekeeper, ABCB1 is widely expressed on the apical membrane in multiple tissues, including liver, kidney, intestine, placenta, and blood-brain barrier [8]. The ABCB1 transporter has identified as a mediator of MDR and catalyzes the transportation of various classes of chemotherapeutic agents, anthracyclines (e.g., daunorubicin and doxorubicin), plant alkaloids (e.g., vincristine and vinblastine), taxanes (e.g., docetaxel and paclitaxel), etc. [8, 9].

T-lymphokine-activated killer cell-originated protein kinase (TOPK), also known as PDZ-binding kinase (PBK), is a Ser/Thr protein kinase of the mitogen-activated protein kinase kinase (MAPKK) family. Functionally, PBK/TOPK performs central roles in regulating cell proliferation, DNA damage repair, cell cycle, apoptosis, immune responses, and inflammation [10]. Based on evidence from The Cancer Genome Atlas database (*gepia.cancer-pku.cn*), the PBK/TOPK expression is remarkably higher in samples from patients with 24 of 31 cancer types compared with in normal tissues. Moreover, according to UALCAN database (*ualcan.path.uab.edu*), 11 of 33 tumor samples show that a high level of PBK/TOPK is linked with survival. Indeed, many studies have been validated that PBK/TOPK overexpression promotes cancer development, malignancy, and poor prognosis in various cancer types, including epithelial ovarian cancer [11], non-small cell lung cancer [12], chordoma [13], gastric carcinoma [14], etc. The findings from Joel et al. [15] have supported the use of PBK/TOPK as a therapeutic target in cancer stem cells-enriched tumors. Importantly, PBK/TOPK is rarely detected in normal tissues with exception for tissues from testis, placenta, and thymus [16, 17]. This highlights the use of PBK/TOPK as a target for cancer therapy and an indicator for cancer prognosis. Interestingly, PBK/TOPK inhibitor labeled with fluorescent or radioactivity can be used for *in vivo* cancer-specific imaging and tumor delineation [18, 19], which further highlights the importance of studies on potential PBK/TOPK inhibitor.

OTS964, as a potent PBK/TOPK inhibitor, strongly suppresses the growth of PBK/TOPK-positive cancer cells in cell-based assays [11, 20], and more importantly, OTS964 promotes complete regression in lung cancer xenograft models [20] and inhibits the growth of patient-derived ovarian cancer cells [11]. In terms of unfavorable hematological toxicity, anemia and leukocytopenia with increased platelets, this effect recovered within 2 weeks and could be circumvented via a liposome-based delivery system [20]. Using [^18^F]FE-OTS964 as a tracer in glioblastoma has proved that OTS964 has a favorable biodistribution and pharmacokinetics, and also, due to the tumor specificity of PBK/TOPK expression, has presented a starting point for developing a noninvasive PBK/TOPK imaging platform, *in vivo* PET imaging, optical imaging, etc. [19]. These findings strongly support the clinical use of OTS964.

In this study, we aimed to comprehensively investigate the relationship between ABCB1 transporter and the regulation of OTS964 efficacy by exploring the transport function, ATPase activity, ligand-receptor interaction, and expression level.

# 2. Material and methods

## 2.1 Reagents, kits, apparatus, and software

Information for used reagents, kits, apparatus, and software are listed in Table S4. Other reagents were purchased from Thermo Fisher Scientific (Waltham, MA) unless mentioned otherwise.

## 2.2 Cell lines and cell culture conditions

The ABCB1-overexpressing MDR cell line KB-C2 was developed and maintained in medium supplemented with 2 μg/ml colchicine (COL) [21]. SW620/Ad300 cells expressing ABCB1 were established and cultured in medium containing 300 ng/ml doxorubicin (DOX) [22]. Their respective drug-sensitive cell lines are human epidermoid carcinoma cell line KB-3-1 and human colon cancer cell line SW620. The *ABCB1* gene knockout subline of SW620/Ad300 and SW620 were constructed using clustered regularly interspaced short palindromic repeats (CRISPR)/CRISPR-associated (Cas) 9 system [23]. HEK293/ABCB1 and HEK293/pcDNA3.1 were transfected with either a pcDNA3.1 vector encoding a full length ABCB1 or an empty vector pcDNA3.1 [24]. ABCB1-knockout and -transfected cell lines were maintained in complete medium in the presence of 1.5 mg/ml and 2 mg/ml G418 (geneticin), respectively. PEL and B1/G2 cells were transfected with a bicistronic vector containing full-length ABCB1 and ABCG2 or an empty vector pcDNA3.1 [25]. Cells co-expressed with ABCB1 and ABCG2 were selected with complete medium with 250 μg/ml zeocin. All cells were cultured in complete medium at 37°C in a humidified incubator supplied with 5% CO_2_. All MDR cells were cultured in drug-free complete medium for at least 3 weeks and passaged for at least 3 generations before further experimental use.

## 2.3 Cell viability assay

The cell viability of chemotherapeutic agents in MDR cell lines and their respective parental cell lines was assessed using an MTT assay. As described previously [26], cells (5,000-7,000 cells/well) were seeded into a 96-well plate and allowed to attach. On the following day, serial concentrations of therapeutic drug were added to the designated wells with or without 2 h pretreatment of modulator at non-toxic concentrations. After a 3-day incubation period, an MTT solution was added and incubated for an additional 3 h. Followed by removing the supernatant, DMSO was added to dissolve the resulting formazan crystals. The OD570 value was measured using a spectrophotometer.

## 2.4 Accumulation assay

The ABCB1-mediated transport function was evaluated using a tritium-labeled paclitaxel (PTX) accumulation assay. Based on an established protocol [27], cells (1×10^6^ cells/well) were seeded into a 24-well plate and allowed to attach. On the following day, modulator at indicated concentrations was added to the designated wells. Following 2 h pretreatment, [^3^H]-PTX was added to designated wells and incubated for an additional 2 h or 72 h, separately. Cells were washed twice with ice-cold PSB, and this was followed by transferring into scintillation fluid. The radioactivity was measured using a liquid scintillation analyzer.

## 2.5 ATPase assay

As previously described [28], the vanadate-sensitive ABCB1 ATPase activity was determined by quantifying the amount of inorganic phosphate (P_i_) produced from hydrolyzed ATP. Briefly, membrane vesicles (10 μg total protein) extracted from High Five insect cells expressing ABCB1 was incubated in assay buffer containing 5 mM sodium azide (NaN_3_), 1 mM ouabain (g-strophanthin), 2 mM dithiothreitol (DTT), 10 mM magnesium chloride (MgCl_2_), 50 mM potassium chloride (KCl), 2 mM ethylene glycol-bis(β-aminoethyl ether)-N,N,N′,N′-tetra acetic acid (EGTA), and 50 mM pH 6.8 2-(N-morpholino) ethanesulfonic acid (MES), with or without 0.3 mM sodium orthovanadate (Na_3_VO_4_), at 37°C for 5 min. Then the mixture was incubated with OTS964 (0-40 μM finial concentration) or OTS964 (0-40 μM finial concentration) with 1 μM tepotinib at the same temperature for 3 min. Mg-ATP (5 mM) solution was then added to initiate a 20-min reaction at 37°C, followed by adding 5% SDS to terminate the reaction. The P_i_ was quantified by measuring the absorbance at 880 nm using a spectrophotometer.

## 2.6 Molecular docking analysis

The 3D structure of OTS964 or PTX was constructed for docking simulation as previously described [29]. Human ABCB1 6QEX (paclitaxel bound) [30] was obtained from RCSB Protein Data Bank (PDB). The human ABCB1 model is inward-facing. Docking calculations were performed in AutoDock Vina (version 1.1.2) [31]. Hydrogen atoms and partial charges were added using AutoDockTools (ADT, version 1.5.4). Docking grid center coordinates were determined from the bound ligands provided in PDB files. Receptor/ligand preparation and docking simulation were performed using default settings. The top-scoring pose (sorted by affinity score: kcal/mol) was selected for further analysis and visualization.

## 2.7 Western blot analysis

The ABCB1 protein expression level was investigated using a Western blot analysis as previously described [32]. Briefly, cells were incubated with OTS964 at indicated concentrations for a serial time-course. Following lysate collection, equal amounts of total proteins were subjected to SDS-PAGE, and then transferred on to a PVDF membrane. The membrane was blocked with 4% non-fat milk, and this was followed by incubation with primary and secondary antibody (1:1000 dilution). The chemiluminescence signal of protein-antibody complex was visualized using ECL substrate and captured using blot scanner as per manufacturer’s instruction. The relative density of each protein band was analyzed by Fiji software.

## 2.8 Quantitative real-time PCR (qRT-PCR)

The ABCB1 mRNA expression level was detected using qRT-PCR based on an established protocol [33]. Total RNA was extracted from cultured cells following designated treatment with RNeasy plus mini kit per manufacturer’s instructions. Total RNA concentrations were quantified at 260 nm with a spectrophotometer. RNA samples with an A260/A280 ratio between 1.8 and 2.0 were used for further analysis. The cDNA was prepared from the extracted RNA in the reverse transcriptase reaction with SuperScript II reverse transcriptase per manufacturer’s protocol. The transcriptome level of ABCB1 transporter was determined by quantitative PCR in an AriaMx real-time PCR system with SYBR select master mix and following specific primer set: ABCB1-Forward: 5’-CTCTTTGCCACAGGAAGCCT-3’, ABCB1-Reverse: 5’-CATTGCGGTCCCCTTCAAGA-3’, GAPDH-Forward: 5’- CTGGGCTACACTGAGCACC-3’, GAPDH-Reverse: 5’-AGTGGTCGTTGAGGGCAATG-3’. The PCR reaction has 40 cycles of 95℃ for 30 s, 55℃ for 1 min, and 72℃ for 1 min. Data were calculated based on the comparative ΔΔC_T_ method and expressed as the relative fold changes.

## 2.9 Experimental animals and xenograft model

Athymic NCR (nu/nu) nude mice (male, 4-5 weeks age) were used for tumor xenograft models. The animals were housed and cared under the St. John’s University animal facility and were monitored closely for tumor growth by palpation and visual examination daily. The protocol for animal experiments was approved by the St. John’s University Institutional Animal Care & Use Committee (IACUC) (Protocol #1984). The research was conducted in compliance with the Animal Welfare Act and other federal statutes.

As previously established by Chen’s laboratory [34], SW620 (5×10^6^ cells/0.2 ml PBS) and SW620/Ad300 (5×10^6^ cells/0.2 ml PBS) cells were injected subcutaneously under armpits of the nude mice. Once the tumor reached approximately 5×5 mm in size, the mice (n = 6 per group) were randomized into the following treatment groups. Group 1 mice were administrated with normal saline intraperitoneally. Group 2 mice were administered with 15 mg/kg OTS964 orally. Group 3 mice were administrated with 10 mg/kg verapamil (VPL) intraperitoneally 1 h before receiving oral gavage of 15 mg/kg OTS964. All treatments were given once daily for a period of 18 days. The OTS964 was prepared in autoclaved water and the VPL was prepared in normal saline. The dosage and administration interval of OTS964 and VPL were selected based on previous studies from Hu et al. [17] and Shen et al. [35] respectively without any remarkable toxicity in mice. It is worthwhile to mention that PBK/TOPK inhibitors (OTS964) cause dysfunction in the differentiation process of hematopoietic stem cells to WBCs and platelets (reduction of WBCs with an increase in platelets) in a dose-dependent manner [20]. According to the results from Matsuo et al. [20], this unfavorable hematopoietic abnormality is observed in nude mice treated with intravenous administration of OTS964, but this is a transient effect in nude mice treated with oral administration of OTS964, as indicated by the spontaneous recovery from leukocytopenia within 2 weeks after treatment stopped. Hence, it is reasonable that oral administration was chosen here, even though hematopoietic toxicity is still a concern and higher dosage should be given by oral gavage. The body weight of mice was measured every 3^rd^ day for monitoring drug dosage. The two perpendicular diameters were recorded every 3^rd^ day and tumor volume was estimated based on the formula: V = (length × width^2^) × 0.5 [26]. Blood was collected in a blood collection tube with heparin to prevent coagulation and to perform blood cell counting. The white blood cells (WBCs) and platelets were counted with WBC diluting fluid and platelet diluent, respectively, as described previously [34]. At the end of the experiment, all mice from each group were euthanized using carbon dioxide, and the tumor tissues were excised, measured, weighed, collected, and stored at -80℃.

## 2.10 Statistics

All data are shown as mean ± SD. Comparisons were made between control group and respective treatment group. The *p* values were computed by one-way or two-way ANOVA followed by Tukey *post hoc* analysis, if appropriate. The *a priori* significance level was *p* < 0.05.

# 3. Study design

MDR in cancer cells is a phenotype whereby cells have attenuated sensitivity to drugs with distinct structures and mechanisms. ABCB1 is a member of the ABC transporter family and involved in MDR. Overexpression of ABCB1 has been reported in both leukemia and solid tumors, facilitating drug efflux to reduce the intracellular level of chemotherapeutic drugs from accumulating in cancer cells [36]. Thus, identifying drugs that are substrates of ABCB1 can provide directions for treatment strategies and improve the quality of cancer patients’ life.

In decade, PBK/TOPK emerges as an attractive cancer-specific therapeutic target and is considered as an oncogenic target or a prognostic marker [10, 16]. As PBK/TOPK is considered as a MAPKK-like protein, it plays a role in MAPK signaling including ERK pathway, p38 MAPK pathway, and JUN pathway, and thus regulates cell proliferation [10, 37]. PBK/TOPK is also involved in PI3K-PTEN-Akt pathway, and thereby promotes tumor development [10, 37]. Interestingly, MAPK signaling and PI3K-Akt signaling are either directly or indirectly linked with regulation of ABCB1 protein expression and/or transcription of *ABCB1* gene [38]. This may support our hypothesis that PBK/TOPK has potential to interact with ABCB1 transporter. OTS964, as a PBK/TOPK inhibitor, suppresses cell proliferation with nanomolar inhibitory potency [39], which is consistent with our results. It has reported that drug resistance to OTS514, an analog of OTS964, can be conferred by ABCB1-overexpressing and antagonized by an ABCB1 inhibitor [40]. In addition, OTS964 was characterized as a substrate of ABCG2 [32], and showed an inhibition toward cyclin-dependent kinase CDK11 activity [39]. Together, PBK/TOPK inhibitors often cause on-target and/or off-target toxicity. Hence, we considered that OTS964 has high possibility to interact with other MDR-associated ABC transporters. In the present study, we focused on the ABCB1-induced acquired resistance to OTS964 and investigated its potential factors.

# 4. References for additional file

1. Siegel RL, Miller KD, Jemal A: **Cancer statistics, 2020.** *CA Cancer J Clin* 2020, **70:**7-30.

2. Keefe DM, Bateman EH: **Tumor control versus adverse events with targeted anticancer therapies.** *Nat Rev Clin Oncol* 2011, **9:**98-109.

3. Mohammad IS, He W, Yin L: **Understanding of human ATP binding cassette superfamily and novel multidrug resistance modulators to overcome MDR.** *Biomed Pharmacother* 2018, **100:**335-348.

4. Eckford PD, Sharom FJ: **ABC efflux pump-based resistance to chemotherapy drugs.** *Chem Rev* 2009, **109:**2989-3011.

5. Levran O, O'Hara K, Peles E, Li D, Barral S, Ray B, Borg L, Ott J, Adelson M, Kreek MJ: **ABCB1 (MDR1) genetic variants are associated with methadone doses required for effective treatment of heroin dependence.** *Hum Mol Genet* 2008, **17:**2219-2227.

6. Dietrich CG, Geier A, Oude Elferink RP: **ABC of oral bioavailability: transporters as gatekeepers in the gut.** *Gut* 2003, **52:**1788-1795.

7. Kalabis GM, Kostaki A, Andrews MH, Petropoulos S, Gibb W, Matthews SG: **Multidrug resistance phosphoglycoprotein (ABCB1) in the mouse placenta: fetal protection.** *Biol Reprod* 2005, **73:**591-597.

8. Szakács G, Váradi A, Ozvegy-Laczka C, Sarkadi B: **The role of ABC transporters in drug absorption, distribution, metabolism, excretion and toxicity (ADME-Tox).** *Drug Discov Today* 2008, **13:**379-393.

9. Germann UA: **P-glycoprotein--a mediator of multidrug resistance in tumour cells.** *Eur J Cancer* 1996, **32a:**927-944.

10. Huang H, Lee MH, Liu K, Dong Z, Ryoo Z, Kim MO: **PBK/TOPK: An Effective Drug Target with Diverse Therapeutic Potential.** *Cancers (Basel)* 2021, **13**.

11. Ikeda Y, Park JH, Miyamoto T, Takamatsu N, Kato T, Iwasa A, Okabe S, Imai Y, Fujiwara K, Nakamura Y, Hasegawa K: **T-LAK Cell-Originated Protein Kinase (TOPK) as a Prognostic Factor and a Potential Therapeutic Target in Ovarian Cancer.** *Clin Cancer Res* 2016, **22:**6110-6117.

12. Shih MC, Chen JY, Wu YC, Jan YH, Yang BM, Lu PJ, Cheng HC, Huang MS, Yang CJ, Hsiao M, Lai JM: **TOPK/PBK promotes cell migration via modulation of the PI3K/PTEN/AKT pathway and is associated with poor prognosis in lung cancer.** *Oncogene* 2012, **31:**2389-2400.

13. Thanindratarn P, Dean DC, Nelson SD, Hornicek FJ, Duan Z: **T-LAK cell-originated protein kinase (TOPK) is a Novel Prognostic and Therapeutic Target in Chordoma.** *Cell Prolif* 2020, **53:**e12901.

14. Ohashi T, Komatsu S, Ichikawa D, Miyamae M, Okajima W, Imamura T, Kiuchi J, Kosuga T, Konishi H, Shiozaki A, et al: **Overexpression of PBK/TOPK relates to tumour malignant potential and poor outcome of gastric carcinoma.** *Br J Cancer* 2017, **116:**218-226.

15. Joel M, Mughal AA, Grieg Z, Murrell W, Palmero S, Mikkelsen B, Fjerdingstad HB, Sandberg CJ, Behnan J, Glover JC, et al: **Targeting PBK/TOPK decreases growth and survival of glioma initiating cells in vitro and attenuates tumor growth in vivo.** *Mol Cancer* 2015, **14:**121.

16. Herbert KJ, Ashton TM, Prevo R, Pirovano G, Higgins GS: **T-LAK cell-originated protein kinase (TOPK): an emerging target for cancer-specific therapeutics.** *Cell Death Dis* 2018, **9:**1089.

17. Hu QF, Gao TT, Shi YJ, Lei Q, Liu ZH, Feng Q, Chen ZJ, Yu LT: **Design, synthesis and biological evaluation of novel 1-phenyl phenanthridin-6(5H)-one derivatives as anti-tumor agents targeting TOPK.** *Eur J Med Chem* 2019, **162:**407-422.

18. Pirovano G, Roberts S, Reiner T: **TOPKi-NBD: a fluorescent small molecule for tumor imaging.** *Eur J Nucl Med Mol Imaging* 2020, **47:**1003-1010.

19. Pirovano G, Roberts S, Brand C, Donabedian PL, Mason C, de Souza PD, Higgins GS, Reiner T: **[(18)F]FE-OTS964: a Small Molecule Targeting TOPK for In Vivo PET Imaging in a Glioblastoma Xenograft Model.** *Mol Imaging Biol* 2019, **21:**705-712.

20. Matsuo Y, Park JH, Miyamoto T, Yamamoto S, Hisada S, Alachkar H, Nakamura Y: **TOPK inhibitor induces complete tumor regression in xenograft models of human cancer through inhibition of cytokinesis.** *Sci Transl Med* 2014, **6:**259ra145.

21. Yoshimura A, Kuwazuru Y, Sumizawa T, Ikeda S, Ichikawa M, Usagawa T, Akiyama S: **Biosynthesis, processing and half-life of P-glycoprotein in a human multidrug-resistant KB cell.** *Biochim Biophys Acta* 1989, **992:**307-314.

22. Lai GM, Chen YN, Mickley LA, Fojo AT, Bates SE: **P-glycoprotein expression and schedule dependence of adriamycin cytotoxicity in human colon carcinoma cell lines.** *Int J Cancer* 1991, **49:**696-703.

23. Lei ZN, Teng QX, Wu ZX, Ping FF, Song P, Wurpel JND, Chen ZS: **Overcoming multidrug resistance by knockout of ABCB1 gene using CRISPR/Cas9 system in SW620/Ad300 colorectal cancer cells.** *MedComm (2020)* 2021, **2:**765-777.

24. Ji N, Yang Y, Cai CY, Lei ZN, Wang JQ, Gupta P, Shukla S, Ambudkar SV, Kong D, Chen ZS: **Selonsertib (GS-4997), an ASK1 inhibitor, antagonizes multidrug resistance in ABCB1- and ABCG2-overexpressing cancer cells.** *Cancer Lett* 2019, **440-441:**82-93.

25. Robinson AN, Tebase BG, Francone SC, Huff LM, Kozlowski H, Cossari D, Lee JM, Esposito D, Robey RW, Gottesman MM: **Coexpression of ABCB1 and ABCG2 in a Cell Line Model Reveals Both Independent and Additive Transporter Function.** *Drug Metab Dispos* 2019, **47:**715-723.

26. Yang Y, Ji N, Cai CY, Wang JQ, Lei ZN, Teng QX, Wu ZX, Cui Q, Pan Y, Chen ZS: **Modulating the function of ABCB1: in vitro and in vivo characterization of sitravatinib, a tyrosine kinase inhibitor.** *Cancer Commun (Lond)* 2020, **40:**285-300.

27. Wu ZX, Yang Y, Teng QX, Wang JQ, Lei ZN, Wang JQ, Lusvarghi S, Ambudkar SV, Yang DH, Chen ZS: **Tivantinib, A c-Met Inhibitor in Clinical Trials, Is Susceptible to ABCG2-Mediated Drug Resistance.** *Cancers (Basel)* 2020, **12**.

28. Yang Y, Ji N, Teng QX, Cai CY, Wang JQ, Wu ZX, Lei ZN, Lusvarghi S, Ambudkar SV, Chen ZS: **Sitravatinib, a Tyrosine Kinase Inhibitor, Inhibits the Transport Function of ABCG2 and Restores Sensitivity to Chemotherapy-Resistant Cancer Cells in vitro.** *Front Oncol* 2020, **10:**700.

29. Wang JQ, Li JY, Teng QX, Lei ZN, Ji N, Cui Q, Zeng L, Pan Y, Yang DH, Chen ZS: **Venetoclax, a BCL-2 Inhibitor, Enhances the Efficacy of Chemotherapeutic Agents in Wild-Type ABCG2-Overexpression-Mediated MDR Cancer Cells.** *Cancers (Basel)* 2020, **12**.

30. Alam A, Kowal J, Broude E, Roninson I, Locher KP: **Structural insight into substrate and inhibitor discrimination by human P-glycoprotein.** *Science* 2019, **363:**753-756.

31. Trott O, Olson AJ: **AutoDock Vina: improving the speed and accuracy of docking with a new scoring function, efficient optimization, and multithreading.** *J Comput Chem* 2010, **31:**455-461.

32. Yang Y, Wu ZX, Wang JQ, Teng QX, Lei ZN, Lusvarghi S, Ambudkar SV, Chen ZS, Yang DH: **OTS964, a TOPK Inhibitor, Is Susceptible to ABCG2-Mediated Drug Resistance.** *Front Pharmacol* 2021, **12:**620874.

33. Zhang GN, Zhang YK, Wang YJ, Gupta P, Ashby CR, Jr., Alqahtani S, Deng T, Bates SE, Kaddoumi A, Wurpel JND, et al: **Epidermal growth factor receptor (EGFR) inhibitor PD153035 reverses ABCG2-mediated multidrug resistance in non-small cell lung cancer: In vitro and in vivo.** *Cancer Lett* 2018, **424:**19-29.

34. Wang YJ, Zhang YK, Zhang GN, Al Rihani SB, Wei MN, Gupta P, Zhang XY, Shukla S, Ambudkar SV, Kaddoumi A, et al: **Regorafenib overcomes chemotherapeutic multidrug resistance mediated by ABCB1 transporter in colorectal cancer: In vitro and in vivo study.** *Cancer Lett* 2017, **396:**145-154.

35. Shen F, Chu S, Bence AK, Bailey B, Xue X, Erickson PA, Montrose MH, Beck WT, Erickson LC: **Quantitation of doxorubicin uptake, efflux, and modulation of multidrug resistance (MDR) in MDR human cancer cells.** *J Pharmacol Exp Ther* 2008, **324:**95-102.

36. Boyer T, Gonzales F, Barthélémy A, Marceau-Renaut A, Peyrouze P, Guihard S, Lepelley P, Plesa A, Nibourel O, Delattre C, et al: **Clinical Significance of ABCB1 in Acute Myeloid Leukemia: A Comprehensive Study.** *Cancers (Basel)* 2019, **11**.

37. Han Z, Li L, Huang Y, Zhao H, Luo Y: **PBK/TOPK: A Therapeutic Target Worthy of Attention.** *Cells* 2021, **10**.

38. Katayama K, Noguchi K, Sugimoto YJNJoS: **Regulations of P-glycoprotein/ABCB1/MDR1 in human cancer cells.** 2014, **2014**.

39. Lin A, Giuliano CJ, Palladino A, John KM, Abramowicz C, Yuan ML, Sausville EL, Lukow DA, Liu L, Chait AR, et al: **Off-target toxicity is a common mechanism of action of cancer drugs undergoing clinical trials.** *Sci Transl Med* 2019, **11**.

40. Stefka AT, Johnson D, Rosebeck S, Park JH, Nakamura Y, Jakubowiak AJ: **Potent anti-myeloma activity of the TOPK inhibitor OTS514 in pre-clinical models.** *Cancer Med* 2020, **9:**324-334.

**
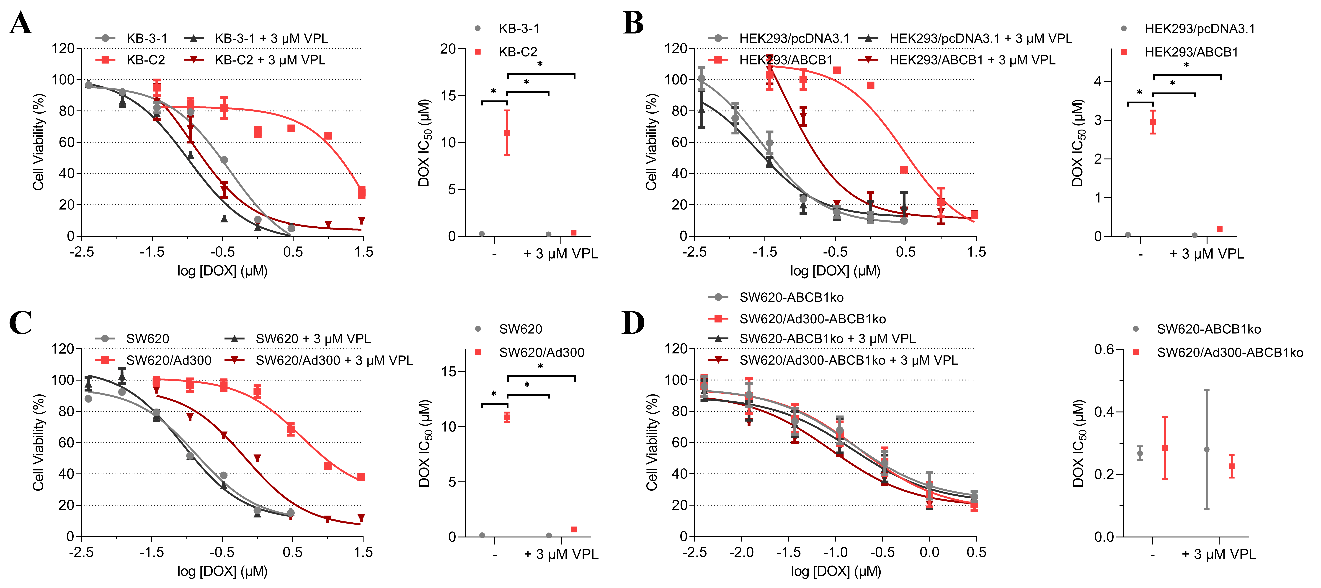
**

Figure S1**. Cytotoxic activity of DOX in drug-selected, gene-transfected, or gene-knockout cells and their respective parental cells.** The concentration-response curves and IC_50_ values for DOX with or without a verified ABCB1 inhibitor in **A)** KB-C2 and KB-3-1, **B)** HEK293/ABCB1 and HEK293/pcDNA3.1, **C)** SW620/Ad300 and SW620, and **D)** SW620/Ad300-ABCB1ko and SW620-ABCB1ko cells. The GraphPad software [log(inhibitor) vs. response] was used to fit nonlinear regression and to calculate IC_50_ values. Each dot is expressed as mean ± SD from a representative of three independent experiments. **p* < 0.05 versus the respective control group.


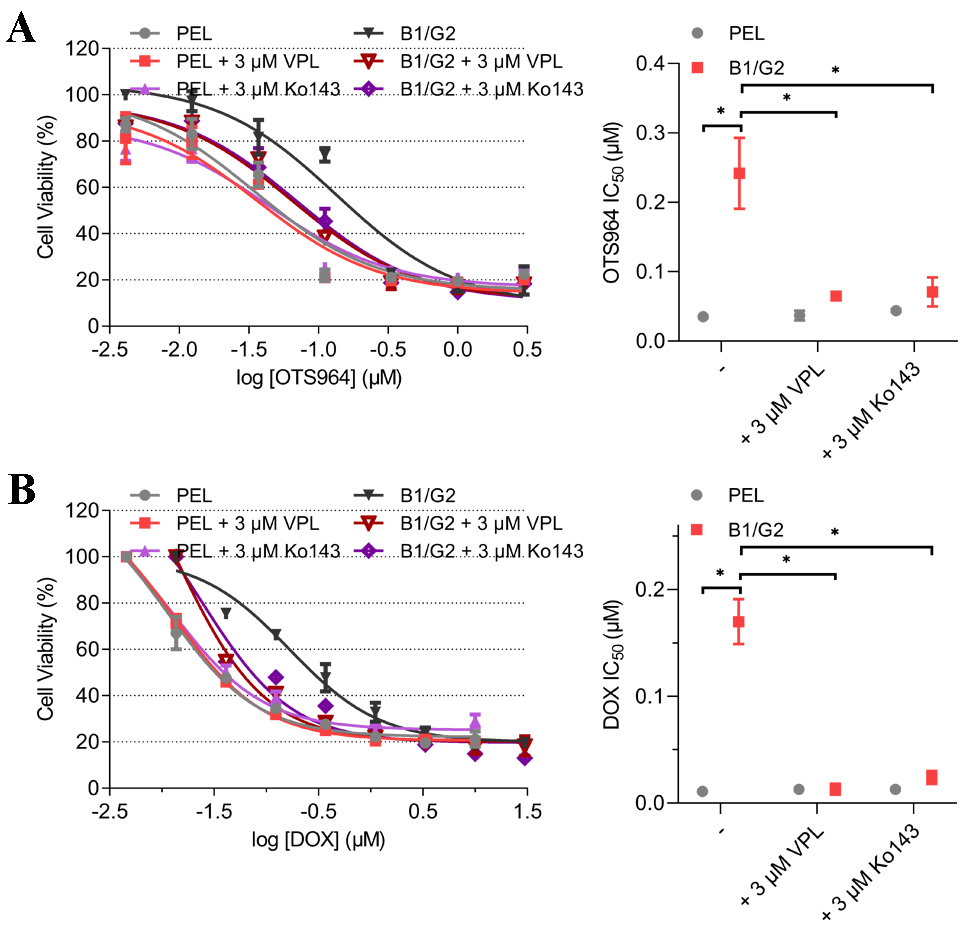


Figure S2**.** **Cytotoxic activity of OTS964 or DOX in cells transfected with both ABCB1 and ABCG2 transporters and its corresponding parental cells.** The concentration-response curves and IC_50_ values for **A)** OTS964 and **B)** DOX with or without a verified ABCB1 or ABCG2 inhibitor in cells transfected with both transporters (B1/G2 cells) and its corresponding parental cells (PEL cells). The GraphPad software [log(inhibitor) vs. response] was used to fit nonlinear regression and to calculate IC_50_ values. Each dot is expressed as mean ± SD from a representative of three independent experiments. **p* < 0.05 versus the respective control group.


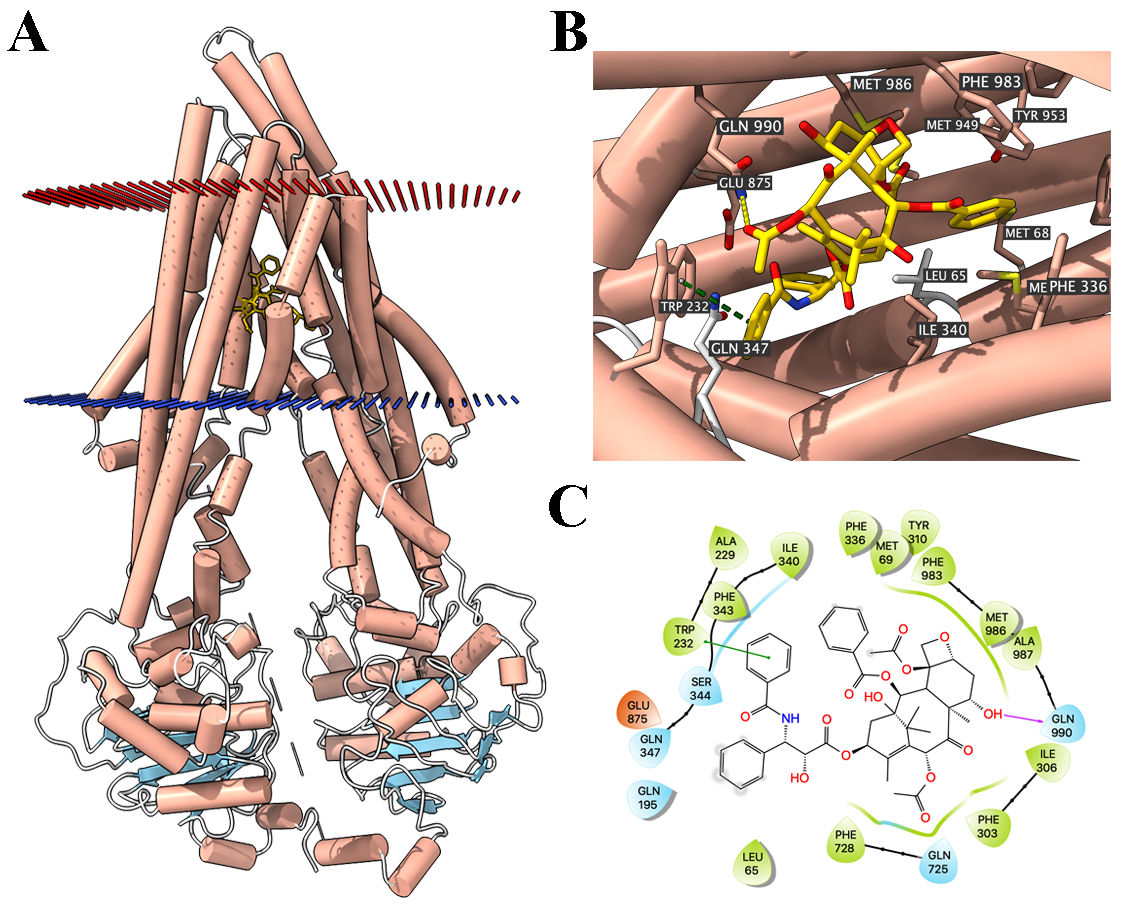


Figure S3**.** **Highest-scoring docked pose of PTX within human ABCB1 at substrate-binding site.** **A)** Overview of the best-scoring pose of PTX in the drug binding pocket of ABCB1 protein. **B)** Details of interactions between PTX and ABCB1 binding pocket. Predicted bonds were displayed as colored dash lines: hydrogen bond: yellow; cation-*pi* interaction: green. **C)** 2D PTX-ABCB1 interaction. Important amino acids were displayed as colored bubbles (green: hydrophobic; blue: polar; red: positively charged). Predicted bonds were displayed as colored lines: green line: *pi*-*pi* stacking; purple line with arrow: hydrogen bond.

Table S1**. The anticancer effectiveness of OTS964 or DOX in drug-sensitive and ABCB1-overexpressing cell lines.**

| Treatment | IC_50_^a^ ± SD (μM) (RF^b^) | | | |
| --- | --- | --- | --- | --- |
|  | KB-3-1 | KB-C2 | HEK293/pcDNA3.1 | HEK293/ABCB1 |
| OTS964 | 0.002 ± 0.002 (1.000) | 0.216 ± 0.073 (137.805) ^*^ | 0.009 ± 0.004 (1.000) | 0.102 ± 0.016 (11.309) ^*^ |
| + VPL 3 μM | 0.002 ± 0.002 (1.481) | 0.039 ± 0.004 (24.899) | 0.008 ± 0.001 (0.907) | 0.010 ± 0.003 (1.063) |
| DOX | 0.276 ± 0.012 (1.000) | 11.053 ± 2.386 (40.053) ^*^ | 0.048 ± 0.008 (1.000) | 2.949 ± 0.296 (61.620) ^*^ |
| + VPL 3 μM | 0.227 ± 0.115 (0.822) | 0.410 ± 0.203 (1.484) | 0.035 ± 0.007 (0.740) | 0.199 ± 0.028 (4.154) |

^a^ IC_50_ values are expressed as mean ± SD from a representative of three independent experiments.

^b^ Resistance fold (RF) was calculated by dividing the IC_50_ values of verified substrate-drugs with or without modulator in drug-sensitive or drug-resistant cells by the IC_50_ values of verified substrate-drugs without modulator in drug-sensitive cells.

^*^*p* < 0.05 versus the respective control group without modulator.

Table S2**. The anticancer effectiveness of OTS964 or DOX in ABCB1-overexpressing and ABCB1-knockout cell lines.**

| Treatment | IC_50_^a^ ± SD (μM) (RF^b^) | | | |
| --- | --- | --- | --- | --- |
|  | SW620 | SW620/Ad300 | SW620-ABCB1ko | SW620/Ad300-ABCB1ko |
| OTS964 | 0.001 ± 0.001 (1.000) | 0.160 ± 0.009 (179.820) ^*^ | 0.044 ± 0.011 (1.000) | 0.047 ± 0.005 (1.056) |
| + VPL 3 μM | 0.001 ± 0.002 (1.312) | 0.006 ± 0.005 (6.931) | 0.058 ± 0.019 (1.300) | 0.039 ± 0.011 (0.891) |
| DOX | 0.163 ± 0.002 (1.000) | 10.845 ± 0.445 (66.452) ^*^ | 0.268 ± 0.022 (1.000) | 0.285 ± 0.099 (1.063) |
| + VPL 3 μM | 0.150 ± 0.013 (0.919) | 0.684 ± 0.029 (4.191) | 0.280 ± 0.190 (1.045) | 0.226 ± 0.036 (0.843) |

^a^ IC_50_ values are expressed as mean ± SD from a representative of three independent experiments.

^b^ Resistance fold (RF) was calculated by dividing the IC_50_ values of verified substrate-drugs with or without modulator in drug-sensitive or drug-resistant cells by the IC_50_ values of verified substrate-drugs without modulator in drug-sensitive cells.

^*^*p* < 0.05 versus the respective control group without modulator.

Table S3**. The anticancer effectiveness of chemotherapeutic drugs with or without modulator in ABCB1-overexpressing cell lines.**

| Treatment | IC_50_^a^ ± SD (μM) (RF^b^) | | | |
| --- | --- | --- | --- | --- |
|  | KB-3-1 | KB-C2 | HEK293/pcDNA3.1 | HEK293/ABCB1 |
| PTX | 0.005 ± 0.001 (1.000) | 1.711 ± 0.351 (346.672) ^*^ | 0.012 ± 0.001 (1.000) | 0.427 ± 0.077 (35.590) ^*^ |
| + OTS964 5 nM | 0.005 ± 0.001 (0.966) | 2.051 ± 0.105 (415.459) ^*^ | 0.010 ± 0.002 (0.795) | 1.079 ± 0.146 (90.017) ^*^ |
| + OTS964 10 nM | 0.006 ± 0.001 (1.163) | 2.261 ± 0.164 (458.110) ^*^ | 0.017 ± 0.001 (1.439) | 1.808 ± 0.108 (150.814) ^*^ |
| + VPL 3 μM | 0.006 ± 0.001 (1.236) | 0.064 ± 0.001 (12.900) | 0.009 ± 0.003 (0.761) | 0.093 ± 0.019 (7.730) |
| DOX | 0.276 ± 0.012 (1.000) | 11.053 ± 1.766 (40.053) ^*^ | 0.048 ± 0.008 (1.000) | 2.949 ± 0.296 (61.620) ^*^ |
| + OTS964 5 nM | 0.331 ± 0.009 (1.198) | 13.835 ± 2.815 (50.136) ^*^ | 0.062 ± 0.020 (1.294) | 3.402 ± 0.310 (71.087) ^*^ |
| + OTS964 10 nM | 0.405 ± 0.053 (1.467) | 13.525 ± 1.082 (49.013) ^*^ | 0.058 ± 0.008 (1.205) | 3.520 ± 0.085 (73.563) ^*^ |
| + VPL 3 μM | 0.227 ± 0.115 (0.822) | 0.410 ± 0.203 (1.484) | 0.035 ± 0.007 (0.740) | 0.199 ± 0.028 (4.154) |
| VCR | 0.003 ± 0.001 (1.000) | 0.597 ± 0.029 (202.238) ^*^ | 0.005 ± 0.001 (1.000) | 0.256 ± 0.027 (52.010) ^*^ |
| + OTS964 5 nM | 0.003 ± 0.001 (0.945) | 0.785 ± 0.080 (266.113) ^*^ | 0.005 ± 0.001 (1.115) | 0.248 ± 0.009 (50.305) ^*^ |
| + OTS964 10 nM | 0.003 ± 0.001 (0.951) | 0.932 ± 0.146 (315.850) ^*^ | 0.004 ± 0.001 (0.829) | 0.281 ± 0.031 (56.953) ^*^ |
| + VPL 3 μM | 0.003 ± 0.001 (1.0681) | 0.040 ± 0.011 (13.607) | 0.005 ± 0.001 (0.964) | 0.063 ± 0.008 (12.700) ^*^ |
| CDDP | 1.232 ± 0.158 (1.000) | 1.568 ± 0.229 (1.273) | 1.048 ± 0.122 (1.000) | 1.084 ± 0.270 (1.035) |
| + OTS964 5 nM | 1.085 ± 0.271 (0.881) | 1.853 ± 0.044 (1.504) | 1.416 ± 0.229 (1.352) | 0.877 ± 0.247 (0.837) |
| + OTS964 10 nM | 1.319 ± 0.175 (1.070) | 1.472 ± 0.643 (1.194) | 1.223 ± 0.035 (1.167) | 1.000 ± 0.227 (0.954) |
| + VPL 3 μM | 1.470 ± 0.436 (1.193) | 1.456 ± 0.083 (1.181) | 1.355 ± 0.447 (1.293) | 1.077 ± 0.012 (1.028) |

^a^ IC_50_ values are expressed as mean ± SD from a representative of three independent experiments.

^b^ Resistance fold (RF) was calculated by dividing the IC_50_ values of verified substrate-drugs with or without modulator in drug-sensitive or drug-resistant cells by the IC_50_ values of verified substrate-drugs without modulator in drug-sensitive cells.

^*^*p* < 0.05 versus the respective control group without modulator.

Table S4. **Information for used reagents, kits, apparatus, and software.**

| Reagents/Kits/Apparatus/Software | Company [location of its headquarters (city, state)] |
| --- | --- |
| OTS964 | ChemieTek (Indianapolis, IN) |
| Fetal bovine serum (FBS) | Atlanta Biologicals (Minneapolis, MN) |
| Antibiotics (penicillin/streptomycin) | Corning (Corning, NY) |
| Dulbecco’s modified Eagle medium (DMEM) |  |
| Trypsin-EDTA |  |
| Eagle’s minimum essential medium (EMEM) | Quality Biological (Gaithersburg, MD) |
| Doxorubicin (DOX) | Medkoo Biosciences (Morrisville, NC) |
| Cisplatin (CDDP) | Enzo Life Sciences (Farmingdale, NY) |
| G418 (Geneticin) |  |
| Ko143 |  |
| Anti-Pgp antibody (F4) | Millipore-Sigma (Burlington, MA) |
| Dimethyl sulfoxide (DMSO) |  |
| Immobilon-P PVDF membrane |  |
| Methylthiazolyldiphenyl-tetrazolium bromide (MTT) |  |
| Paclitaxel (PTX) |  |
| Verapamil (VPL) |  |
| Vincristine (VCR) |  |
| HRP-conjugated secondary antibody | Cell Signaling Technology (Dancers, MA) |
| Anti-GAPDH antibody (GA1R) | Thermo Fisher Scientific (Waltham, MA) |
| Liquid scintillation cocktail |  |
| Pierce™ BCA protein assay kit |  |
| SuperScript™ II reverse transcriptase |  |
| SYBR™ select master mix |  |
| Zeocin (100 mg/ml in HEPES) |  |
| [^3^H]-Paclitaxel (26.1 Ci/mmol) | Moravek Biochemicals (Brea, CA) |
| ABCB1 primer | Eurofins Genomics (Louisville, KY) |
| RNeasy plus mini kit | QIAGEN (Germantown, MD) |
| Microvette^®^ 500 capillary blood collection tubes | VWR Chemicals (Radnor, PA) |
| Eng Scientific WBC diluting fluid | Fisher Scientific (Fair Lawn, NJ) |
| Eng Scientific platelet diluent |  |
| Fisherbrand™ accuSkan™ GO UV/Vis microplate spectrophotometer |  |
| Packard TRI-CARB^®^ 1900CA liquid scintillation analyzer | Packard Instrument (Downers Grove, IL) |
| C-DiGit^®^ blot scanner | LI-COR Biotechnology (Lincoln, NE) |
| Eppendorf biospectrometer | Eppendorf North America (Hauppauge, NY) |
| AriaMx real-time PCR system | Agilent Technologies (Santa Clara, CA) |
| Fiji software for Windows | NIH (Bethesda, MD) |
| GraphPad Prism software version 8.3.0 for Windows | GraphPad Software (La Jolla, CA) |
| Athymic NCR (nu/nu) nude mice (male, 4-5 weeks age) | Taconic Farms (Albany, NY) |
